# Histopathologic, immunophenotypic, and proteomics characteristics of low-grade phyllodes tumor and fibroadenoma: more similarities than differences

**DOI:** 10.1038/s41523-020-0169-8

**Published:** 2020-06-26

**Authors:** Lingxin Zhang, Chen Yang, John D. Pfeifer, Richard M. Caprioli, Audra M. Judd, Nathan H. Patterson, Michelle L. Reyzer, Jeremy L. Norris, Horacio M. Maluf

**Affiliations:** 1 Department of Pathology & Immunology, Washington University School of Medicine, St. Louis, MO 63110 USA; 2 Department of Biochemistry, Vanderbilt University School of Medicine, Nashville, TN 37235 USA; 3 Present Address: Department of Pathology, Memorial Sloan Kettering Cancer Center, New York, NY 10065 USA; 4 Present Address: Department of Pathology, Cedars Sinai Medical Center, Los Angeles, CA 90048 USA

**Keywords:** Breast cancer, Surgical oncology, Outcomes research

## Abstract

Distinguishing low-grade phyllodes tumor from fibroadenoma is practically challenging due to their overlapping histologic features. However, the final interpretation is essential to surgeons, who base their management on the final pathology report. Patients who receive a diagnosis of fibroadenoma might not undergo any additional intervention while lumpectomy with wide margins is the standard of care for phyllodes tumor, which can have significant cosmetic consequences. We studied the clinical, immunophenotypic, and proteomics profiles of 31 histologically confirmed low-grade phyllodes tumor and 30 fibroadenomas. Matrix-assisted laser desorption ionization (MALDI) imaging mass spectrometry (IMS) and immunohistochemistry for Ki-67, p53, β-catenin, and E-cadherin were performed on all cases. After the mass spectra for all 31 cases of low-grade phyllodes tumor and 30 cases of fibroadenoma were collected, an average peak value for all cases was generated. There was no significant difference in the overall mass spectra pattern in any of the peaks identified. There was also overlap in the percentage of cells staining positive for Ki-67, p53, β-catenin, and E-cadherin. The two groups of patients showed no statistically significant difference in age, tumor size, or disease-free survival. Neither group developed malignant transformation, distant metastases, or disease-related mortality. We have demonstrated low-grade phyllodes tumor and fibroadenoma to show significant overlapping clinical and proteomics features.

## Introduction

Fibroepithelial lesions of the breast are a heterogeneous group of biphasic tumors that include the common benign fibroadenomas and the relatively rare phyllodes tumors (2.5% of all fibroepithelial lesions of the breast). Current treatment guidelines rely on histologic diagnosis on either core or excisional biopsy and recommend wide excision without axillary staging to treat phyllodes tumors regardless of grade^1^. However, phyllodes tumor represents a heterogenous group of neoplasms that display highly variable prognosis^2^.

Many grading systems for phyllodes tumor have been proposed, all based on the assessment of tumor borders, stromal cellularity and atypia, mitotic activity, and stromal overgrowth. The World Health Organization (WHO)^2^ recommended a three-tier grading system (benign, borderline, malignant), while the Armed Forces Institute of Pathology (AFIP) proposed a two-tier system (low-grade, high-grade)^3^ with slight variation in their cutoff criteria. AFIP defines low-grade phyllodes tumors as fibroepithelial neoplasms with well-defined borders, uniform leaf-like processes, rare to moderate mitoses (<3 mitoses per 10 high-power fields), mildly increased cellularity often with subepithelial accentuation, no to mild cytological atypia and no sarcomatous stromal overgrowth. The low-grade category proposed by the AFIP would include the majority of benign phyllodes tumors and a subset of borderline phyllodes tumors.

**Fig. 1:**
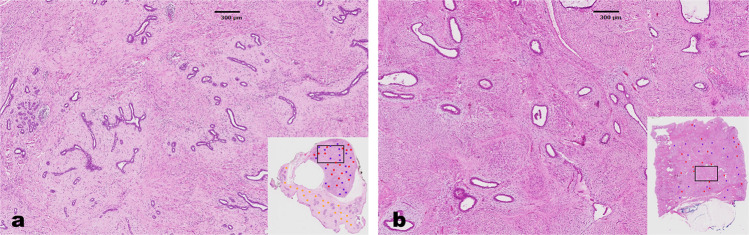
Comparison of histologic features of fibroadenoma and low-grade phyllodes tumor. Histologic features of fibroadenoma (**a**, 20×) and low-grade phyllodes tumor (**b**, 20×). Note that there is significant histologic overlap between the two entities.

Despite the above well-established diagnostic criteria, distinguishing low-grade phyllodes tumor from fibroadenoma is challenging due to their overlapping histologic features (Fig. 1). Recent study by Lawton et al.^4^ showed that there is significant inter-observer variability, even among pathologists who specialize in breast pathology, when making diagnosis of either benign phyllodes tumor or fibroadenoma in a research setting. The issue is more significant in small core biopsies, which determines whether the fibroepithelial lesion should be observed (fibroadenoma) or excised with a wide margin (phyllodes tumor)^5^. The justification for the latter is on the basis of reported local recurrences of phyllodes tumors^6^. This diagnostic dilemma on core needle biopsies has been highlighted during recent attempts of incorporating digital pathology into routine practice^7^. To date, there are no clinically validated immunohistochemical assays to facilitate the differential diagnosis.

The application of matrix-assisted laser desorption ionization (MALDI) imaging mass spectrometry (IMS) on formalin-fixed, paraffin-embedded tissue allows for the label-free, multiplex analysis of thousands of analytes across the tissue surface and elucidate both the localization and relative abundance of endogenous metabolites, lipids, peptides, and proteins^8^. Since the development of histology-directed MALDI-IMS technique^8^, there have been several feasibility studies exploring its utility in the facilitation of challenging histologic diagnoses^9–11^. One main advantage of histology-directed MALDI-IMS is the ability to interrogate the proteomics profile of individual tumor components. This approach has definite appeal in our case, as both phyllodes tumors and fibroadenomas are biphasic tumors, it would be of significant interest to compare the epithelial and stromal components between the two biphasic lesions independently.

The aim of this study was to perform a comprehensive evaluation of the clinical, histopathologic, immunophenotypic, and proteomics characteristics of low-grade phyllodes tumor and fibroadenoma to exploit similarities and differences of these two entities.

## Results

### Clinical characteristics

**Fig. 2:**
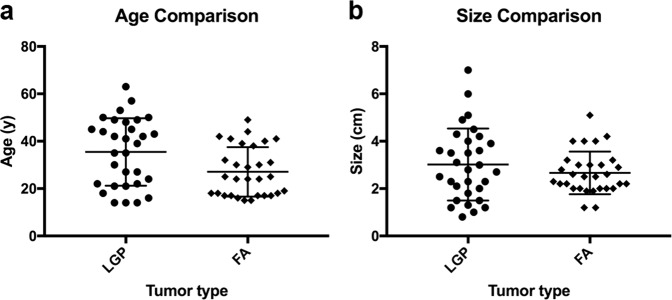
Comparison of patient age and tumor size distribution of low-grade phyllodes tumor (LGP) and fibroadenoma (FA). Dot plot of individual data points, with a horizontal line at the arithmetic mean and error bars showing plus and minus one standard deviation, shows significant overlap in patient age (**a**) and tumor size (**b**) between LGP and FA. Wilcoxon-Mann–Whitney tests show no statistical difference in distribution of patient age or tumor size between the two groups.

A total of 31 patients with low-grade phyllodes tumor (including 22 cases of benign and 9 cases of borderline phyllodes tumors with archived materials available for analysis in our institution, Supplementary Table 1) and 30 patients with fibroadenoma were included in our analysis. All patients were female. There was a slight trend that show low-grade phyllodes tumor to be more commonly seen in older patients with a median age of 39.0 years and a mean age of 35.5 years (range 14 to 63 years), compared to patients with fibroadenoma who had a median age of 24.5 years and a mean age of 27.0 years (range 15 to 49 years). However, difference in age was not statistically significant (Fig. 2a). There was also no statistically significant difference in the size of low-grade phyllodes tumor and fibroadenomas (Fig. 2b). Low-grade phyllodes tumors had a median size of 2.8 cm and a mean size of 3.0 cm (range 0.8 to 7.0 cm) at the time of resection, only marginally larger than the median size of 2.5 cm and mean size of 2.7 cm (range 1.2 to 5.1 cm) of fibroadenomas at the time of surgery.

**Fig. 3:**
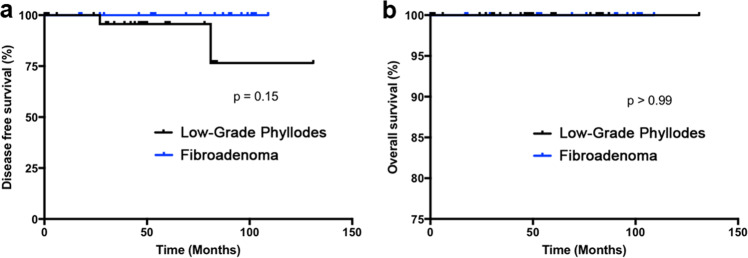
Kaplan–Meier analysis does not show any significant difference in disease-free survival or overall survival in patients with fibroadenoma and low-grade phyllodes tumor. Of note, the two cases of recurrent low-grade phyllodes tumor presented at the previous resection sites, and no recurrences were reported after the re-excision.

On clinical follow-up, 24 of 31 (77.4%) cases with the diagnosis of low-grade phyllodes tumor had re-excision of the tumor bed performed, whereas none of the cases with the diagnosis of fibroadenoma undergone additional intervention. This is consistent with the current standard of care. The median follow-up time for low-grade tumor was 47.0 months (mean 44.6 months, range 0 to 131 months), compared to the 61.5 months median follow-up time for fibroadenoma (mean 59.2 months, range 0 to 109 months). Two of 31 patients (6.5%) with low-grade phyllodes tumor subsequently developed recurrence of disease, and upon a second excision procedure, there were no further recurrences. Although none of the 30 patients with fibroadenoma developed “recurrences,” it is important to note that one patient presented with new ipsilateral breast mass, one patient with contralateral breast mass, and one patient with bilateral breast masses. None of which were biopsied under the clinical impression that these were fibroadenomas. The disease-free Kaplan–Meier curve is presented to show no significant difference in Fig. 3a. There were no malignant transformation, distant metastasis, or mortality related to disease in the low-grade phyllodes tumor or fibroadenoma group. The Kaplan–Meier curve for overall survival is shown in Fig. 3b.

### Immunohistochemistry

**Fig. 4:**
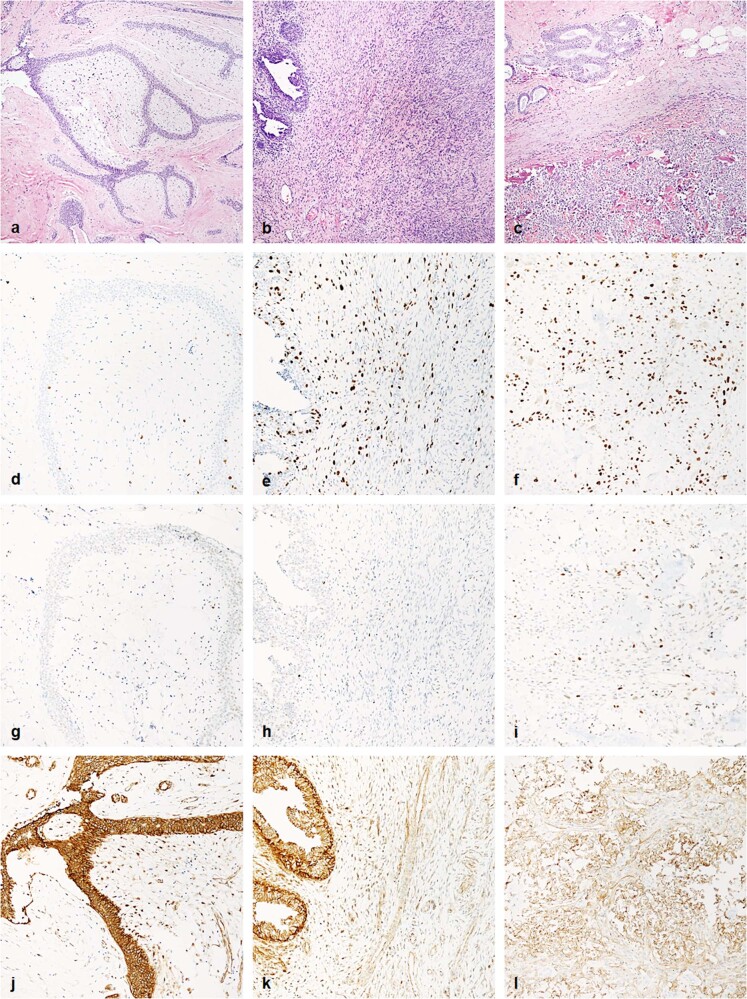
Representative histologic and immunohistochemical images of fibroadenoma, low-grade phyllodes tumor, and high-grade phyllodes tumor. Fibroadenoma (left column), low-grade phyllodes tumor (middle column), and high-grade phyllodes tumor (right column) stained with hematoxylin and eosin (**a**–**c**, 100×), Ki-67 (**d**–**f**, 200×), p53 (**g**–**i**, 200×), and β-catenin (**j**–**l**, 200×).

**Fig. 5:**
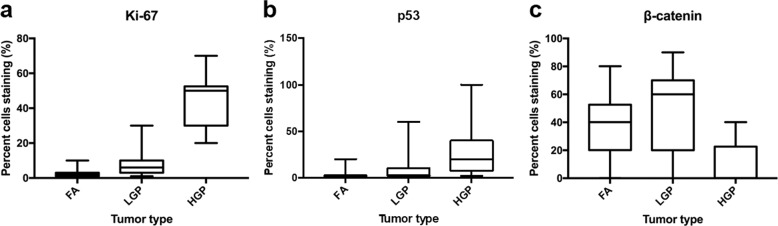
Box and whisker plot of immunohistochemistry results. Percentage of tumor stromal cells staining positive for immunohistochemistry markers Ki-67 (**a**), p53 (**b**), and β-catenin (**c**) in fibroadenoma (FA), low-grade phyllodes tumor (LGP), and high-grade phyllodes tumor (HGP) is plotted. Center line represents median percentage of tumor stromal cells staining positive for each marker; the lower bound of box represents first quartile; the upper bound of box represents third quartile; and whiskers extends to the lowest and highest staining percentage of each marker. Note there is overlap in immunohistochemistry staining in the three entities. Wilcoxon-Mann–Whitney tests show statistically significant difference (*p* < 0.05) in Ki-67 and p53 staining from FA to LGP to HGP. There is no difference in β-catenin staining between FA and LGP, but there is statistically significant difference in nuclear staining for HGP compared with FA and LGP.

There is significant overlap in the percentage of cells staining positive for Ki-67, p53, β-catenin, and E-cadherin between low-grade phyllodes tumor and fibroadenoma (Figs. 4 and 5). Ki-67 had a median positivity of 6.0% (mean 7.7%, range 2 to 30%) in low-grade phyllodes tumor, higher than the 2.0% (mean 2.5%, range 1 to 10%) seen in fibroadenoma. p53 had a median positivity of 3.0% (mean 10.0%, range 1 to 60%) in low-grade phyllodes tumor, which was also higher than the 1.0% (mean 2.7%, range 1 to 20%) seen in fibroadenoma. β-catenin had a median positivity of 60.0% (mean 48.8%, range 2 to 90%) in low-grade phyllodes tumor, again slightly overexpressed, compared to the 40.0% (mean 38.2%, range 0 to 80%) seen in fibroadenoma. However, this was not statistically significant. E-cadherin demonstrated no staining in any stromal cells while exhibiting 100% strong positivity in the epithelial component in both low-grade phyllodes tumors and fibroadenomas.

In contrast, high-grade phyllodes tumor showed minimal overlap and statistically significantly different staining pattern for Ki-67, p53, and β-catenin (Figs. 4 and 5) compared with either low-grade phyllodes tumor group or fibroadenoma group. Ki-67 highlighted a median proliferation index of 50.0% (mean 45.0%, range 20 to 70%), p53 showed a median staining of 20.0% (mean 30.5%, range 2 to 100%), and β-catenin had a median staining of 0% (mean 9.5%, range 0 to 40%). E-cadherin showed the same pattern as low-grade phyllodes and fibroadenomas and was completely negative in the stromal tumor cells.

The results of the immunohistochemistry study are summarized in Table 1.

**Table 1:** Mean percentage of tumor stromal cells positive for immunohistochemistry marker.

	Fibroadenoma (A)	Low-grade phyllodes tumor (B)	High-grade phyllodes tumor (C)	*p-*value A vs. B	*p*-value A vs. C	*p-*value B vs. C
Ki-67	2.5%	7.7%	45.0%	<0.001	<0.001	<0.001
p53	2.7%	10.0%	30.5%	<0.001	<0.001	0.01
β-catenin	38.2%	48.8%	9.5%	0.099	<0.001	<0.001
E-cadherin	0	0	0	1	1	1

### MALDI imaging mass-spectrometry

**Fig. 6:**
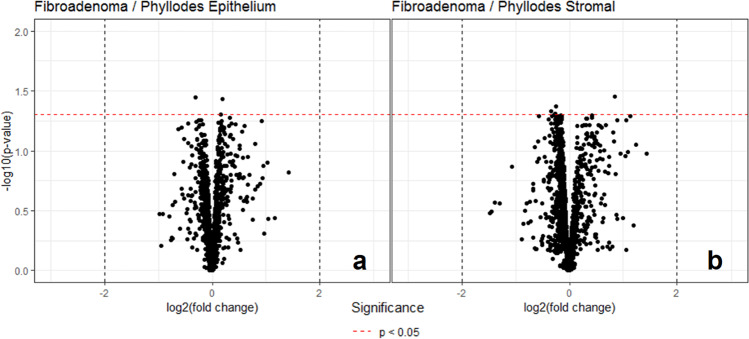
Volcano plots for peak intensity comparison of the epithelial and stromal components of fibroadenoma and low-grade phyllodes tumor, respectively, sampled by matrix-assisted laser desorption ionization (MALDI) imaging mass spectrometry (IMS). Each black point on the plot is an individual *m/z*. The *x*-axis of the plot shows the fold change in peak intensity where data points on the left indicate higher peak intensity in fibroadenoma and data points on the right indicate higher peak intensity in phyllodes tumor. The areas between the two vertical black dotted lines indicated less than minimal change. The *y*-axis shows the *p*-value resulting from an unpaired *t*-test of fibroadenoma vs. low-grade phyllodes tumor samples with the horizontal red dotted line indicating a significant threshold of 0.05 in *p-*value. The volcano plot shows wider distribution of the data points in the stromal component (**b**) than in the epithelial component (**a**) indicating more difference in the stromal component between the two entities. However, the data points are below the *p* < 0.05 threshold.

**Fig. 7:**
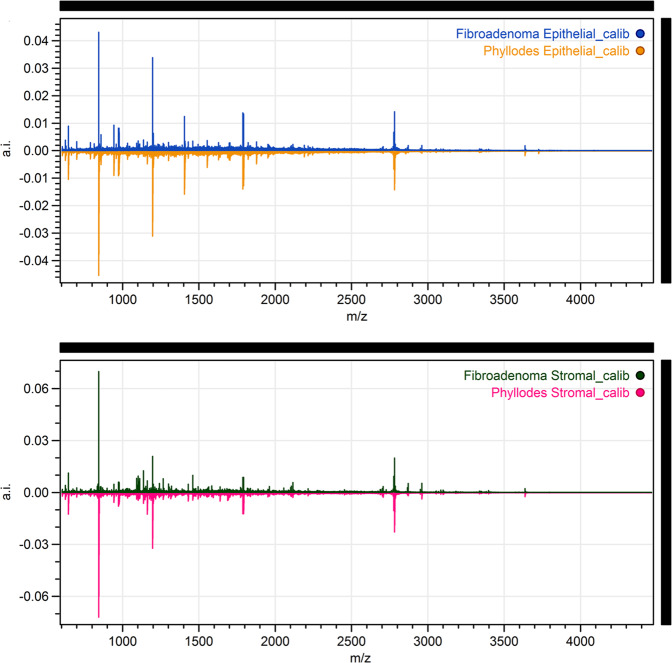
Mean spectra from the fibroadenoma and low-grade phyllodes tumor matrix-assisted laser desorption ionization (MALDI) imaging mass spectrometry (IMS) data. Mean spectra were generated from normalized, recalibrated data. Result from epithelial component is shown on top and the result from the stromal component is shown on the bottom. The *x*-axis of the plot is *m/*z whereas the *y*-axis is the mean spectra. The bottom spectra (phyllodes tumor) in each plot are arbitrary flipped to make comparison easier. As shown, there is no visually discernable difference in the mean spectra of fibroadenoma and low-grade phyllodes tumor.

A total of 21 cases of low-grade phyllodes tumor and another 20 cases of fibroadenoma were randomly selected as the study set to generate an algorithm to distinguish low-grade phyllodes tumor and fibroadenoma. This algorithm was then tested on a validation set of remaining ten cases of low-grade phyllodes tumor and fibroadenoma. The initial mass spectra generated from the study set showed only few peaks with >1-fold difference and fewer peaks that showed statistical significance. There is greater proteomic variability between the stromal compared to the epithelial component in these two tumor types (Fig. 6). However, when the algorithm is applied to the validation set, only 10 (50%) cases were correctly identified by the algorithm (8 of 10 low-grade phyllodes tumors and 2 of 10 fibroadenomas). Only 7 (35%) cases were consistently identified as either phyllodes tumor or fibroadenoma across all annotated areas, while the remaining cases showed 60–90% consistency in different areas of the tumor, suggesting significant tumor heterogeneity in both the fibroadenoma and low-grade phyllodes tumor cases. The seven cases included two fibroadenomas that were identified as low-grade phyllodes tumor by the algorithm. After the mass spectra for all 31 cases of low-grade phyllodes tumor and 30 cases of fibroadenoma are collected, an average peak value for all cases was generated. There is no significant difference in any of the peaks identified, and the overall mass spectra pattern is shown in Fig. 7.

## Discussion

Distinguishing a fibroadenoma from a low-grade phyllodes tumor by pure morphology can be challenging, particularly on core needle biopsies. Our study shows that, even when strictly adhering to the diagnostic criteria, there is minimal difference in the clinical characteristic and overall outcome between low-grade phyllodes tumor and fibroadenoma and that perhaps separating the two entities is neither possible nor desirable. Low-grade phyllodes tumor are slightly larger in size and present in older patients, but neither age or size differences are statistically significant. We also demonstrated that low-grade phyllodes tumor have a low risk for recurrence and malignant potential on long term follow-up, which is similar to findings in other studies in the literature^12^. Within the study cohort, the local recurrence rate of low-grade phyllodes tumor was 6.5%, all of which were cured by re-excision of the tumor bed. Whether this represented incomplete excision of the initial surgery, or rather a recurrence of the tumor could not be further discerned in the retrospective data. We did confirm that the margin status was all negative on evaluation of initial resection. We also noted, however, that although there were no reported “recurrences” for fibroadenomas, new lesions were seen in the breast on mammography, and were often not biopsied due to minimal risk of progression. There were no malignant transformation or distal metastases in any of the low-grade phyllodes tumor or fibroadenoma cases. In the three largest studies to date, only 0.3% of low-grade phyllodes tumor eventually developed metastatic disease^13–15^.

We reviewed the clinical outcome for high-grade phyllodes tumor and 2 of the 8 (25.0%) patients with available follow-up information died of metastatic disease 18 and 19 months after surgery, respectively. This number is slightly above the 16.3% rate reported in the literature^13–15^, likely due to the limited number of patients with high-grade phyllodes tumor in our study. This highlights the importance of distinguishing high-grade phyllodes tumor from low-grade phyllodes tumor, which do show significant prognostic differences.

There are no established criteria for evaluation of immunohistochemistry stains to help classify or stratify fibroadenoma and phyllodes tumor. In our study, Ki-67 showed lowest proliferative index in fibroadenomas (median 2.0%, mean 2.5%, range 1 to 10%), followed by low-grade phyllodes tumor (median 6.0%, mean 7.7%, range 2 to 30%), with high-grade phyllodes tumor showing the highest proliferation index (median 50.0%, mean 45.0%, range 20 to 70%). This is similar to results from previous studies, which some also found high proliferation index to be associated with worse clinical outcome^16–18^. The difference between fibroadenomas and low-grade phyllodes tumor may be explained by a bias to designate as phyllodes tumor those lesions with increased stromal mitotic figures. Previous studies have shown p53 positivity to be increased in phyllodes tumors^16–18^. We also observed the lowest positivity in fibroadenomas (median 1.0%, mean 2.7%, range 1 to 20%), second low-grade phyllodes tumor (median 3.0%, mean 10.0%, range 1 to 60%), followed by high-grade phyllodes tumor (median 20.0%, mean 30.5%, range 2 to 100%). β-catenin nuclear staining has been used as a surrogate marker for *Wnt* signaling pathway, which have been proposed to play a role in the pathogenesis of phyllodes tumor^19^. β-catenin had highest nuclear positivity in low-grade phyllodes tumor (median 60.0%, mean 48.8%, range 2 to 90%), followed by fibroadenoma (median 40.0%, mean 38.2%, range 0 to 80%), with high-grade phyllodes tumor rarely expressing the marker (median 0%, mean 9.5%, range 0 to 40%). The loss of nuclear β-catenin expression in high-grade phyllodes tumor has been shown in previous studies and correlate with worse prognosis^20^. Given the overlap in immunohistochemistry results for Ki-67, p53, and β-catenin, it would seem unlikely that these markers would provide much additional information to help distinguish fibroadenoma and phyllodes tumor. There was one study showing E-cadherin in the stromal cells to correlate with recurrence and shorter tumor-specific survival^21^, however, we did not identify any staining for E-cadherin in the stromal component of any of our cases. This difference may be attributed to the alternative antibody clone used in our study.

Although immunohistochemistry studies have the advantage of examining protein expression in the context of histomorphology, the main caveat to this approach is that only selected proteins of interest can be studied at a time. The choice of proteins that can be studied is also restricted by the antibodies that are available. Conventional mass-spectrometry can be utilized to study multiple peptides, lipids, and other molecules, but lacks the capability of spatial recognition. MALDI-IMS provides a platform with spatial specificity to separately compare the stromal and epithelial components between fibroadenoma and low-grade phyllodes tumor. To our knowledge, our study is the first to look at the proteomics constitution of the stromal and epithelial component of low-grade phyllodes tumor and fibroadenoma separately.

Our results showed no significant difference in proteomic profile of the stromal or the epithelial component between low-grade phyllodes tumor and fibroadenoma, as the algorithm generated from the mass spectra in the study set did not confidently distinguish the two entities in the validation set. Furthermore, the combined average of all the peaks generated for the mass spectra showed no significant difference between the two entities. Clinically, none of the low-grade phyllodes tumor or the fibroadenoma cases in our study developed distant metastasis on follow-up and by the fact that a total of five cases developed subsequent lesions in the breast, two cases were low-grade phyllodes tumor which were cured of disease on re-resection, and the other three were cases of fibroadenoma and the new lesions were not biopsied, likely a consequence of interpreting new lesions preceded by a diagnosis of phyllodes tumor as a recurrence and those by a diagnosis of fibroadenoma as multicentricity. It seems that a propensity for recurrence/multicentricity cannot be accurately outlined by known histologic criteria nor by the histology-directed mass spectral protein profiles. Given the limited number of cases included in our study and the low number of proteins analyzed, further investigation is warranted.

In the last two decades, the utility of next generation sequencing has provided new insights in the classification and pathogenesis of breast fibroepithelial lesions. The most important and well-established finding has been the identification of hotspot mutations in exon 2 of *MED12*, an X-linked gene encoding mediator complex subunit 12, in fibroadenomas and phyllodes tumors^22–28^, in the stromal components^22,25,26^. Mutational analysis in several cohorts showed higher rate of *MED12* exon 2 hotspot mutations in fibroadenomas and lower grade phyllodes tumors, particularly benign phyllodes tumors^27,29–31^, while genetic aberrations in *TP53, RB1, NF1, PIK3CA, ERBB4*, and *EGFR* were more commonly found in malignant phyllodes tumors^23,31–33^. Pareja et al.^34^ reported a higher frequency of *MED12* mutations in phyllodes tumors with fibroadenoma-like areas, compared with those without fibroadenoma-like areas, suggesting the progression of *MED12*-mutant fibroadenomas to a subset of phyllodes tumors. Tan et al.^31^ used combined whole exome sequencing and targeted deep sequencing of fibroepithelial lesions and revealed similar genetic signatures between fibroadenomas and a subset of benign phyllodes tumors. For diagnostic purposes, there have been proposal of using an assay of five-gene transcript set (*ABCA8*, *APOD*, *CCL19*, *FN1*, and *PRAME*) for classifying breast fibroepithelial lesions into fibroadenomas and phyllodes tumors^35^.

Most recent consensus^12^ acknowledged that the definitive distinction between cellular fibroadenoma and benign phyllodes tumor may not be crucial and indeed not necessary. We further showed that conventional fibroadenoma and low-grade phyllodes tumor (which also includes borderline phyllodes tumor) are identical by proteomics analysis. Overall, the results from our study as well as those of focusing on genetic aspects support the interpretation these lesions are one and the same and a more conservative surgical approach is justified by its indolent nature, something that can be facilitated by avoiding the diagnosis of phyllodes tumor, at least for those lesions designated as benign phyllodes and those in which a distinction between fibroadenoma and phyllodes tumor is difficult or not possible. In any case of uncertainty, especially in core biopsies, rendering the diagnosis of “benign fibroepithelial tumor” is preferable. Separating out the high-grade (or malignant) phyllodes tumor, on the other hand, has significant clinical implications. There are strict histologic criteria and immunohistochemistry studies that add supporting evidence to this distinction.

There were several limitations to our study that need to be acknowledged. First, our study was retrospective in nature, and was limited by the inherent bias associated with such approach. Second, the number of cases in our study was relatively small. This is mainly due to the fact that phyllodes tumor are much less common in the area populated with Caucasians and African Americans. Third, we did not include specific information on laterality and location of the masses, which may provide additional information in predicting availability for intervention and/or tumor behavior. Lastly, we did not have enough cases of phyllodes tumor with recurrence and/or metastasis to perform MALDI-IMS, which would be of clinical relevance to differentiate from other cases. Additional prospective studies with larger cohorts, especially with large number of cases with recurrences and/or metastases, would be necessary to fully address the issues above.

## Methods

### Case selection

Our study was approved by the Washington University School of Medicine Institutional Review Board, including a waiver of consent given the retrospective nature of this study. The surgical pathology archives were searched for partial or total mastectomies with a diagnosis, including the word “phyllodes” from year 2006 to 2014. Archived histologic slides of these cases and of a matching number of fibroadenomas were retrieved and reviewed by three pathologists (C.Y., L.Z., and H.M.) blinded to the clinical outcome information. All tumors were subclassified using both the WHO^2^ and AFIP^3^ grading criteria. Fibroadenoma variants, including juvenile fibroadenoma, cellular fibroadenoma, myxoid fibroadenoma, and complex fibroadenoma with florid changes that obscure the underlying fibroadenomatous nature were excluded due to potential challenges in accurate classification. Fibroadenomas with focal complex changes were not excluded. A total of 31 cases with confirmed diagnosis of low-grade phyllodes tumor and 30 consecutive cases of fibroadenoma were identified and retrieved from archives. Clinical data for all cases of low-grade phyllodes tumor and fibroadenoma were extracted from the electronic medical records system for analysis. We additionally analyzed all ten cases of high-grade phyllodes tumor identified with immunohistochemistry using Ki-67, p53, β-catenin, and E-cadherin.

### Immunohistochemistry

One representative formalin-fixed paraffin-embedded tissue block from each case (31 low-grade phyllodes tumor, 30 fibroadenoma, and 10 high-grade phyllodes tumor) was selected to generate 4 μm thick unstained slides for immunohistochemical staining for the following antibodies: Ki-67 (clone 30-9, prediluted, Ventana), p53 (clone Bp53-11, prediluted, Ventana), β-catenin (clone 14, prediluted, Ventana), and E-cadherin (clone EP700Y, prediluted, Ventana). The immunohistochemistry staining was performed on Ventana Benchmark automated immunostainer (Ventana Medical Systems, Inc., Tucson, AZ) according to standard protocols with appropriate positive and negative controls. The immunohistochemistry staining results were interpreted individually by two pathologists (C.Y. and L.Z.) blinded of the diagnosis. The percentage of cells positive was visually estimated and recorded and the average of the two results were used for analysis. Only nuclear staining for Ki-67, p53, and β-catenin in the stromal tumor cells were considered as positive. In contrast, cytoplasmic and membranous staining in the stromal tumor cells for E-cadherin was interpreted as positive. The percentage of staining was estimated relative to total number of stromal cells.

### MALDI-IMS

**Fig. 8:**
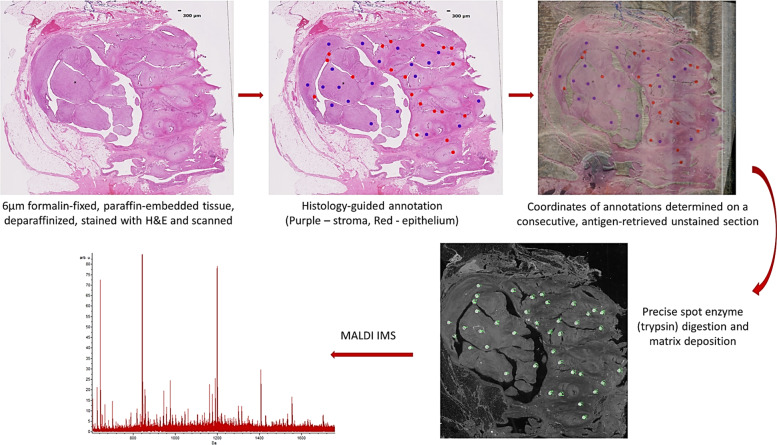
Procedure of matrix-assisted laser desorption ionization (MALDI) imaging mass spectrometry (IMS). Two serially sectioned 6 μm sections from formalin-fixed paraffin-embedded tissue blocks are generated. One slide is stained with hematoxylin and eosin and scanned for annotation. After the slide is annotated by the pathologists, the areas of interest are mapped on the unstained slide. After enzyme digestion and matrix application, the mass spectra of individual annotated areas are obtained.

Serial sections, 6 μm thick, were cut from the above selected blocks. One section per sample was mounted onto indium tin oxide coated slide for MALDI time-of-flight (TOF) analysis, whereas the consecutive section was mounted onto a regular glass slide as a reference histologic section. The slides were dried overnight at 37 °C and stored out of light until ready to use. The reference slides were stained with H&E and scanned with an automated slide scanner (Leica SCN 400, Leica Biosystems, Buffalo, Illinois) at x20 magnification and uploaded to a web-based pathology interface for mass-spectrometry (PIMS)^36^. Two pathologists (L.Z. and C.Y.) then annotated the scanned images with color-coded circular marks (300 μm in diameter). In each image of fibroadenoma or phyllodes tumor, 20 marks (red circles) were assigned for the epithelial component and 20 marks (purple circles) were assigned for the stromal components (preferably adjacent to the epithelial lumina) (Fig. 8). In a subset of cases, surrounding uninvolved breast stroma was annotated as reference (yellow circles). Using image-processing software (Photoshop), the histology-annotated image was merged with an image of the consecutive unstained section and the coordinates of the annotations were determined. Serial sections for MALDI TOF were deparaffinized and antigen retrieved. Briefly, slides were deparaffinized in a series of xylenes, graded ethanol and water washes and dried completely at room temperature. Antigen retrieval was performed in a Coplin jar containing 10 mM Tris buffer, pH 9, in a decloaking chamber. The samples were brought to 95 °C in the decloaking chamber for 20 min, cooled to 90 °C for 10 s and removed from the chamber. The slides were cooled in the Coplin jar for 20 min prior to buffer exchange with Milli-Q purified water. The slides were dried at room temperature and stored over desiccant out of light. Trypsin and matrix were applied to the discrete regions selected in PIMS using a Labcyte Portrait 630 robotic spotter with a spotting error <60 µm^37^. Trypsin (0.08 µg/µL solution) was deposited over a series of 40 iterations, 40 passes, one drop each pass (roughly 170 pL/drop) at each designated coordinate to achieve a final concentration of ~29 ng/mm^2^. This was completed over a total of 2 h to allow drying time between iterations. Alpha-cyano-4-hydroxycinnamic acid matrix (5 mg/ml in 50% acetonitrile, 0.1% trifluoroacetic acid) was then applied over the digested spots over 72 iterations, 8 passes, 9 drops each pass. The samples were analyzed in profiling mode on a Bruker UltrafleXtreme TOF MS in reflectron positive mode. Data was sorted by patient and tissue type.

Acquired MALDI-IMS profiling data was processed using MALDIquant^38^ in the R environment. After data import, the spectra were baseline corrected to remove MALDI chemical noise using the SNIP algorithm^39^ with 20 iterations. After baseline correction, the spectral data was normalized by the total ion current to account for pixel-to-pixel signal artifacts. Following normalization, the spectral data was statistically re-aligned to a reference spectrum to remove inherent mass shifts from the TOF instrument that can greatly affect classification quality. The reference spectrum for realignment was closest spectrum to the 50th quantile of the TIC distribution of all data. Following mass axis alignment, peaks with signal-to-noise ratio above 3 were selected on the overall mean spectrum from all data. The peak intensity data was integrated for each spectrum resulting in a data matrix where observations (rows) are individual pixels labeled by de-identified patient serial number and pathologist annotation and variables (columns) are picked peak intensity data from the dataset from each spectrum.

Machine learning on the picked peak MS data implemented the support vector machine algorithm with a linear kernel. The model and cost function (regularization) were tuned using 5-fold cross validation. The tuned cost function was found to be 0.5 in this instance. Analysis of significant different peaks between the two data classes was performed using area under the receiver operator characteristic curve.

### Statistics

Relationships between the variables were examined by Wilcoxon-Mann–Whitney tests for comparison of means, Fisher-Exact tests for comparison of categorical variables, and log-rank tests for comparison of survival. All tests were two-sided.

### Reporting summary

Further information on research design is available in the Nature Research Reporting Summary linked to this article.

## Supplementary information

Supplementary Table 1.

Reporting Summary Checklist

## Data Availability

Raw data were generated at the Washington University School of Medicine, a large-scale facility. A metadata record describing the data supporting this article can be found in the figshare repository with 10.6084/m9.figshare.12264725^40^. The proteomics data have been deposited in the MassIVE repository under accession code MSV000085409^41^. Clinical characteristics are included in the supplementary information of this article, as Supplementary Table 1. Overview data of the comparison between phyllodes tumor and fibroadenoma can be found in the file Phyllodes Data.xlsx, which is included with the metadata record^40^.

## Code availability

We used the caret package in R for machine learning^42^ along with unmodified preprocessing algorithms from MALDIquant mentioned in the manuscript.
