# Supplementary material for: Histopathologic, immunophenotypic, and proteomics characteristics of low-grade phyllodes tumor and fibroadenoma: more similarities than differences
Source: NPJ Breast Cancer. 2020 Jun 26;6:27. doi: 10.1038/s41523-020-0169-8 (PMC7319981; doi:10.1038/s41523-020-0169-8)
Supplement: Supplementary file 1 — Supplementary Table 1. [file 41523_2020_169_MOESM1_ESM.pdf]

Supplementary Table 1. Clinicopathologic features and follow-up of low-grade phyllodes tumors

Age (y)	Size (cm)	AFIP 2-tier diagnosis	WHO 3-tier diagnosis	Follow-up
57	4	Low-grade	Benign	Re-excision negative
43	1.8	Low-grade	Borderline	Re-excision negative
50	6	Low-grade	Borderline	Re-excision negative
49	3.5	Low-grade	Benign	Re-excision negative
35	3.5	Low-grade	Benign	Re-excision negative
35	7	Low-grade	Benign	Re-excision positive; recurred in 3.5 years
18	3.9	Low-grade	Benign	Re-excision negative
30	4.9	Low-grade	Borderline	Re-excision negative
42	2.8	Low-grade	Borderline	Re-excision negative
45	2	Low-grade	Benign	Re-excision negative
16	2.7	Low-grade	Benign	Re-excision positive
22	4.3	Low-grade	Borderline	Re-excision negative
53	4.5	Low-grade	Borderline	Re-excision negative
21	2.3	Low-grade	Benign	Re-excision negative
27	0.8	Low-grade	Benign	Re-excision negative
44	1.3	Low-grade	Benign	Re-excision negative
49	1.2	Low-grade	Benign	Re-excision negative
22	2.3	Low-grade	Benign	No recurrence
50	2.3	Low-grade	Benign	No recurrence
45	1.5	Low-grade	Benign	No recurrence
21	2.5	Low-grade	Benign	Re-excision negative
14	5.1	Low-grade	Benign	No recurrence
42	2.1	Low-grade	Benign	Re-excision negative
41	4.2	Low-grade	Borderline	Re-excision negative
48	1.5	Low-grade	Benign	Re-excision negative
63	1.2	Low-grade	Borderline	No recurrence
27	1	Low-grade	Benign	No recurrence
39	3.1	Low-grade	Benign	Re-excision positive
14	3	Low-grade	Benign	Re-excision positive; recurred in 2 years
24	3.6	Low-grade	Benign	Re-excision negative
14	3.6	Low-grade	Borderline	No recurrence
